# Cervical Spinal Alignment Change Accompanying Spondylosis Exposes Harmonization Failure with Total Spinal Balance: A Japanese Cohort Survey Randomly Sampled from a Basic Resident Registry

**DOI:** 10.3390/jcm10245737

**Published:** 2021-12-08

**Authors:** Shota Ikegami, Masashi Uehara, Ryosuke Tokida, Hikaru Nishimura, Noriko Sakai, Hiroshi Horiuchi, Hiroyuki Kato, Jun Takahashi

**Affiliations:** 1 Department of Orthopaedic Surgery, Shinshu University School of Medicine, 3-1-1 Asahi, Matsumoto 390-8621, Nagano, Japan; sh.ikegami@gmail.com (S.I.); horiuchih@aol.com (H.H.); hirokato@shinshu-u.ac.jp (H.K.); jtaka@shinshu-u.ac.jp (J.T.); 2 Rehabilitation Center, Shinshu University Hospital, 3-1-1 Asahi, Matsumoto 390-8621, Nagano, Japan; tryosuke@shinshu-u.ac.jp (R.T.); hikaru5@shinshu-u.ac.jp (H.N.); 3 Department of Orthopaedic Surgery, New Life Hospital, Obuse, Nagano 381-0295, Kamitakai-gun, Japan; wpowaro@yahoo.co.jp

**Keywords:** epidemiological study, resident cohort, resident registry, spinal alignment, spinal balance, cervical spine, aging, gender, adult spine

## Abstract

The relationship between spinal posture and quality of life has garnered considerable attention with the increase in older community-dwelling residents. However, details of this association remain insufficient. A recent Japanese population cohort epidemiological locomotion survey (the Obuse study) revealed that the C2–C7 cervical sagittal vertical axis (CSVA) began to increase in males from their 60s, but not in females. This study aimed to clarify the pathology of these cervical spondylotic changes. A total of 411 participants (202 male and 209 female) aged between 50 and 89 years were selected by random sampling from a cooperating town’s resident registry. All participants underwent lateral X-ray photography in a standing position for the measurement of several sagittal spinal alignment parameters, including CSVA, C2–C7 cervical lordosis (CL), T1 slope (T1S), and sagittal vertical axis (SVA). The presence of cervical spondylotic changes was also recorded. Associations of cervical sagittal spinal alignment with cervical spondylosis and between cervical and total sagittal spinal alignment were examined. The prevalence of cervical spondylosis was significantly higher in males (81%) than in females (70%) (*p* = 0.01). CL was significantly smaller in cervical spondylosis subjects when adjusted by age (3.4 degrees less; *p* = 0.01). T1S minus CL displayed a moderate positive correlation with CSVA in both males and females (r = 0.49 and 0.48, respectively, both *p* < 0.01). In males only, CSVA and CL showed weak positive correlations with SVA (r = 0.31 and 0.22, respectively, both *p* < 0.01) independently of age. Cervical spinal misalignment was more clearly associated with diminished SF-8^TM^ scores in females than in males. In community-dwelling elderly residents, cervical sagittal spinal alignment change accompanying cervical spondylosis manifested as hypofunction to compensate for whole-spine imbalance.

## 1. Introduction

Sagittal spinal alignment is more strongly correlated than coronal spinal alignment to health-related quality of life (HRQOL) [1] even for mild spinal deformity, which can be an important barometer of health status for ordinary citizens. Sagittal spinal alignment deteriorates with age in community-dwelling older people [2,3,4]. In addition, an increase in sagittal vertical axis (SVA; anteriorization of the center of gravity line of the cervical spine base) is associated with a decrease in lumbar lordosis [5]. Age-related changes in lumbo-pelvic condition are known to affect sagittal spinal alignment. Two epidemiological studies conducted in different regions corroborated the finding of a characteristic gender difference in the process of spinal alignment change with aging. Specifically, alignment changes over time in males were prominent in the cervical spine region, while females predominantly displayed changes in the lumbo-pelvic area [3,4]. However, no clear evidence has been presented on the reasons for such phenomena. Spinal alignment can be affected by a variety of factors, including activity level and profession. Age-related cervical spondylosis may contribute to poor alignment of the cervical spine [6]. This study aimed to clarify the pathomechanism of sagittal cervical alignment changes in community-dwelling older residents.

## 2. Materials and Methods

### 2.1. Creation of a Randomly Sampled Resident Cohort for Epidemiological Survey

In the establishment of a new population study of Japanese people, we employed random sampling from the basic resident registry of a cooperating town to minimize selection bias and obtain a cohort representative of the general population. Residents between the age of 50 and 89 years were randomly sampled from the basic resident registry of a town to construct a 415-participant cohort termed “the Obuse study” cohort. “Obuse” is the name of the cooperating town located in the central inland area of Japan, with a population of approximately 10,000 people. The Obuse study is a comprehensive investigation on the locomotion health of community-dwelling older people. In the Obuse study cohort, 411 individuals who were able to stand unassisted and whose cervical spinal alignment could be measured were subjected to analysis. According to participant interview results, 21 subjects with a history of thoracolumbar spondylosis and five with a history of rheumatoid arthritis were included. As they were not in such a condition that would cause them to lose their standing balance, they were added to the analysis. Individuals with spinal instrumentation surgery were not included, and those with diagnosed illnesses that significantly altered balance, such as adult spinal deformity and Parkinson’s disease, were excluded as well.

### 2.2. X-ray Examination and Measurement of Spinal Alignment

All participants underwent lateral X-ray photography for the measurement of sagittal spinal alignment parameters, including C2–C7 sagittal vertical axis (CSVA; the distance between a plumb line from the center of the C2 vertebral body and posterior superior corner of C7), C2–C7 cervical lordosis (CL; the angle between the C2 inferior endplate and C7 inferior endplate), T1 slope (T1S), and SVA. The average values of measurements by two board-certified spine surgeons and a trained staff member were used for each parameter. The inter-rater reliability of each parameter was as follows: 0.96 for CSVA, 0.88 for CL, 0.88 for T1S, and 0.95 for SVA. The presence of cervical spondylotic changes was also recorded. The two spine surgeons independently determined the presence or absence of spondylotic changes, with cases determined as having spondylosis by both raters being regarded as spondylotic (inter-rater reliability: 0.95). Osteophyte formation around the vertebral endplates with a loss of intervertebral disc height as well as osteophyte formation and osteosclerotic change of the articular facet joints were defined as spondylotic changes.

### 2.3. HRQOL Assessments

SF-8™ Health Survey measures were determined for all participants for HRQOL evaluation. Results were calculated and expressed as two summary scores: physical component summary (PCS) and mental component summary (MCS).

### 2.4. Statistical Analysis

We compared cervical sagittal spinal alignment parameters between spondylotic and non-spondylotic groups using linear regression models. The response variable was the alignment parameter and the explanatory variables were the presence of cervical spondylosis and age. The Pearson correlation coefficient between T1S minus CL, which is also known as the residual lordotic compensation for subcervical anterior tilting [7], and CSVA were assessed for each gender. We examined the correlation between cervical and subcervical alignment parameters following age adjustment for each gender. For other analyses, Welch’s t-test was used to compare quantitative variables, and Fisher’s exact test was used to compare qualitative variables. Statistical analyses were carried out using the statistical package R, version 3.4.3 (available at http://www.r-project.org accessed on 26 November 2021). The level of significance was set at *p* < 0.05.

## 3. Results

**Figure 1:**
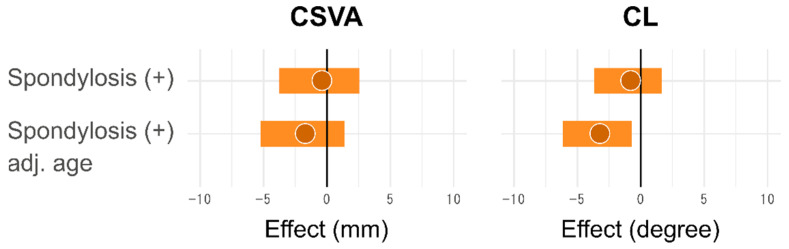
Impact of cervical spondylosis on cervical alignment parameters. Note: Bands represent 95% confidence interval. Abbreviations: CSVA, cervical sagittal vertical axis; CL, cervical lordosis; adj. age, multivariate analysis adjusted by age.

Table 1 shows the baseline characteristics of the Obuse study cohort. The 411 participants were almost uniformly divided into every gender/age decade category. Tertiary industry workers represented the majority of Obuse town residents in their 50s, although this proportion decreased for subjects in their 60s, likely due to mandatory retirement. Table 2 shows the spinal alignment distributions by gender. Overall CSVA and T1S were significantly larger in males (both *p* < 0.01), with no remarkable gender differences for CL or SVA (*p* = 0.54 and *p* = 0.96, respectively). The prevalence of cervical spondylosis in males and females was 80.7% and 69.9% respectively (Table 2). Cervical spondylotic change was significantly more frequent in males (*p* = 0.01, Fisher’s exact test). There were no remarkable differences for CSVA or CL in subjects with or without spondylosis. The odds ratios, 95% confidence intervals, and *p*-values for spinal parameters were as follows: CSVA, −0.6 (−3.8, 2.5), *p* = 0.70; and CL, −1.0 (−3.7, 1.6), *p* = 0.45. CL became significantly smaller in subjects with cervical spondylosis when adjusted by age (−3.4 (−6.1, −0.7), *p* = 0.01) (Figure 1).

**Figure 2:**
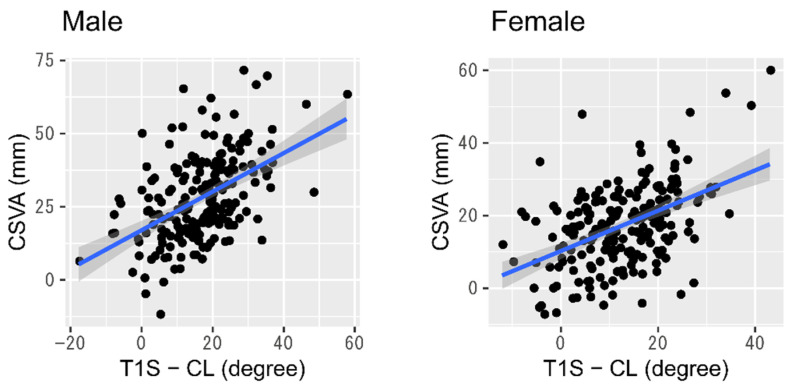
Relationship between cervical anteriorization and T1S minus CL. Abbreviations: CSVA, cervical sagittal vertical axis; T1S, T1 slope; CL, cervical lordosis.

**Figure 3:**
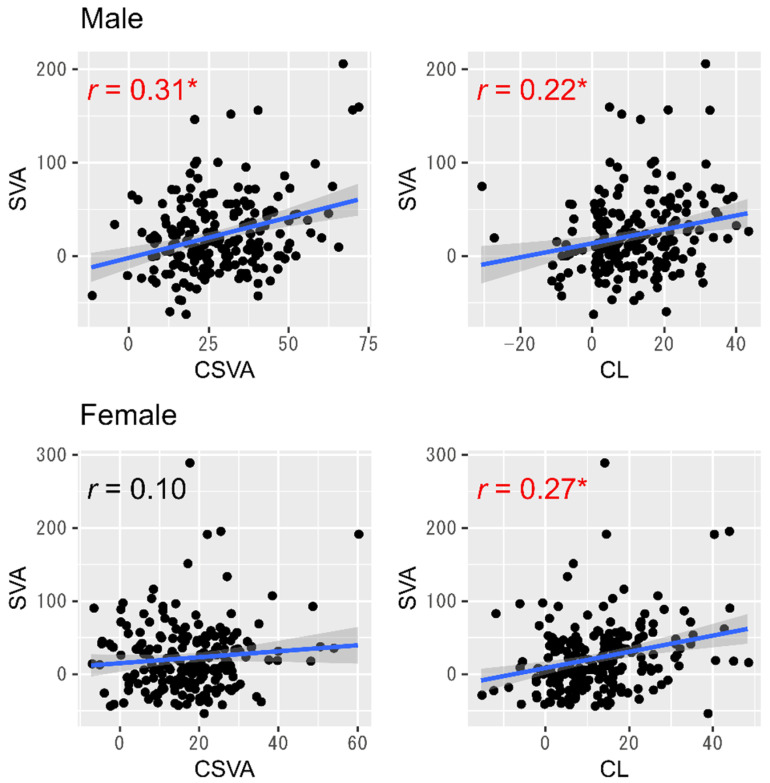
Relationship between subcervical alignment and cervical alignment parameters. Note: * denotes a significant association (*p* < 0.05). Abbreviations: SVA, sagittal vertical axis; CSVA, cervical sagittal vertical axis; CL, cervical lordosis.

T1S minus CL displayed a significant moderate positive correlation with CSVA in both genders (Pearson correlation coefficient: 0.49 for males and 0.48 for females, both *p* < 0.01) (Figure 2). Only in males, however, did both CSVA and CL show mild positive correlations with SVA independently of age (Figure 3 and Table 3).

Table 4 and Table 5 summarize how cervical and subcervical spinal alignment impacted HRQOL. Specifically, larger SVA was significantly associated with a lower PCS score in both genders independently of age. CSVA associated significantly with PCS score in females only, which was independent of age. Larger T1S minus CL was also significantly related to lower PCS scores after adjustment for age in women, with no clear association between cervical spinal alignment and HRQOL in men (Table 4). No remarkable associations were observed for cervical or subcervical spinal alignment among MCS scores (Table 5).

## 4. Discussion

This study revealed that male community-dwelling elderly residents more frequently exhibited cervical spondylotic changes than female residents did, but without subaxial lordosis compensating for the anterior tilting of the subcervical spine. This non-compensation resulted in axis anteriorization accompanying deteriorated subcervical alignment. Thus, cervical sagittal spinal alignment deteriorations with cervical spondylosis may manifest as a compensatory function for diminished whole-spine balance rather than solely as a consequence of spinal degeneration.

**Figure 4:**
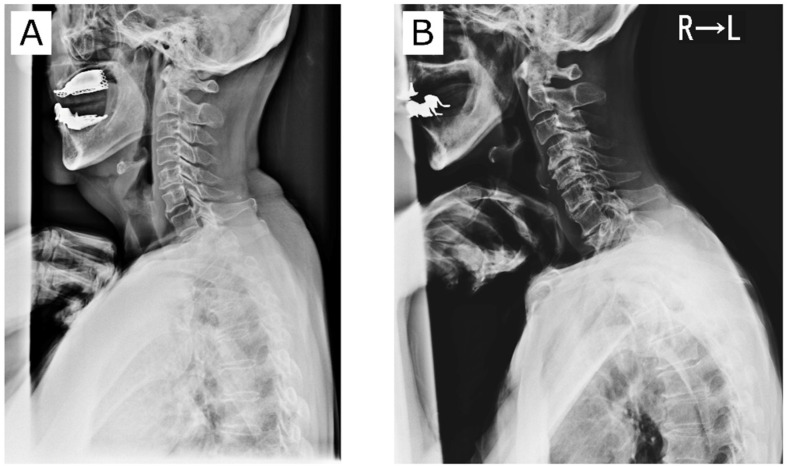
Effect of cervical spondylotic changes on cervical spine alignment. Notes: Case (**A**) (female) has a compensated cervical spine. Case (**B**) (male) has a decompensated cervical spine.

The following is a possible pathomechanism of cervical decompensation, especially in males. First, SVA and T1S increase with aging [4]. However, there is insufficient lordotic compensation due to a range of motion decrease along with a higher prevalence of cervical spondylosis [6]. This leads to decompensated axis anteriorization. The cervical spine has variable normal morphology [8]. One author reported that SVA and T1S were important in determining cervical alignment [9]. A large T1S requires a correspondingly higher CL to preserve sagittal balance. Even in cervical laminoplasty patients, T1S is one of the most important factors determining postoperative cervical spinal alignment [10,11,12]. Figure 4 contrasts representative cervical spine alignment conditions. Cases A and B had virtually identical T1S. In Case A (female), CL suitable for T1S was formed such that the position of the center of gravity of the head was optimized and the front gaze posture was preserved. On the other hand, Case B (male) had obvious cervical spondylotic change and was unable to achieve CL suitable for T1S. As a result, the head has shifted anteriorly. Based on the results of this study, the A-type cervical spine may be less susceptible to changes in subcervical alignment, while the B-type spine may tend to situate more anteriorly due to its susceptibility to subcervical alignment.

Larger values of either T1S minus CL or CSVA have been associated with low HRQOL condition in adult spinal deformity patients [13,14]. These cervical spine alignment parameters were also associated with the Neck Disability Index in cervical operation patients [15,16,17]. The subjects in our study were residents and not spinal deformity or cervical operation patients. Nevertheless, as with subcervical alignment, T1S minus CL and CSVA were significantly associated with HRQOL. It was noteworthy that these relationships between alignment parameters and HRQOL in cervical spine surgery patients were present even in pre-disease populations. However, such associations were significant only in females in our cohort. The reason for this gender difference is unclear and requires further examination. The effect size is small and may be irrelevant given the size of the effect.

Another study of 50–89-year-old Japanese residents (the TOEI study) showed that cervical deformity (i.e., CSVA ≥ 40 mm) residents had significantly lower HRQOL index scores [18]. Although the results in females agreed with our own, those for males did not. This could have been due to differences in the prevalence of cervical deformity in the target population; the TOEI study had cervical deformity prevalences of 31% for male and 9% for female, which were 19% and 2% respectively in this study and significantly lower (*p* < 0.01, Fisher’s exact test). Our earlier studies revealed that the change in spinal alignment with aging in males first appears in the cervical spine and that an age-related increase in CSVA was not noticeable in females [4]. On the other hand, larger CSVA was associated with physical performance deterioration [19]. Insufficient physical performance affects HRQOL, even in healthy local residents [20]. Cervical spine anteriorization in females may occur in a low physical tolerance condition as compared to males.

Lastly, it is difficult to ascertain a direct causal relationship between mental health and spinal posture, and their precise association remains unclear. Although this study found no significant relationship between the factors, there have been reports of a link of recurring depressive episodes to poor spinal posture [21]. Health status is holistic, and clinically useful associations may be identified in the future for mental health and spinal posture.

The limitations of the current investigation include the possibility of inter-observer bias. The high concordance rate was proof that the evaluation was legitimate, but the possibility of bias risk could be further reduced by adding the evaluations of radiologists from a different specialty. As this research was cross-sectional, the direction of the causal relationship between spinal alignments could not be specified. Longitudinal surveys are needed to obtain a definitive conclusion on aging-dependent changes. In this non-compulsory survey, the proportion of people randomly sampled who were ultimately enrolled was less than one third, with 882 people refusing to participate, implying incomplete selection bias and participation bias elimination. Furthermore, no a priori calculations were made regarding a sample size that would ensure sufficient clinical variation to support conclusions that could be generalized; thus, the findings could have been influenced by the composition and prevalence of spondylosis and spinal deformity of the patients who agreed to participate. Regional characteristics were also a limitation of this study in that we sampled subjects from a relatively small town. Although the benefits of recruiting in such regions are lower resident displacement and greater ease in performing an epidemiological survey, the results may differ from those of urban-dwelling residents. Moreover, we could not prove the absence of selection bias by presenting the results of a cohort in one town. Previous papers [4] have shown that the spinal alignment status of Obuse residents was comparable to that from other parts of Japan, implying no particular physical characteristics in our test group, at least among the Japanese. On the other hand, it is very likely that other ethnic groups have different physical characteristics, and so further study is needed in other populations. This study analyzed the relationship among age, gender, and spinal alignment. Spinal alignment can also be affected by a variety of other factors, including activity level and profession. These will be addressed in future studies to deepen our findings. The mechanism of female alignment change could also not be ascertained in this report. Cervical spinal alignment change is more likely to occur in males, but the effects of changes in the cervical spine on HRQOL are more apparent in females. We suspect that cervical alignment is not linearly related to HRQOL, and that females with poor alignment may be subject to lower HRQOL than males with similar findings. Longitudinal studies on this point are needed.

## 5. Conclusions

In conclusion, cervical sagittal spinal alignment changes accompanying spondylosis in the general elderly population manifested as hypofunction to compensate for whole-spine imbalance. Men have a higher prevalence of cervical spondylosis, and their inflexible cervical spine has difficulty compensating for subcervical alignment deterioration. This is likely why males are more prone to large CSVA as a sagittal spinal misalignment. In contrast, cervical spinal misalignment was more clearly associated with low HRQOL in females.

## Figures and Tables

**Table 1:** Baseline characteristics of the study cohort.

Gender	Age (Years)	Number	Height (cm)	Weight (kg)	BMI (kg/m^2^)	Job (Pri; Sec; Ter; None)
Male	50s	50	171.8 (6.0)	67.1 (9.1)	22.7 (2.9)	3; 7; 40; 0
	60s	53	166.7 (4.7)	66.9 (7.7)	24.1 (2.7)	18; 5; 19; 11
	70s	54	163.1 (5.0)	59.9 (10.3)	22.4 (3.5)	22; 2; 7; 23
	80s	45	160.1 (5.7)	57.5 (8.5)	22.4 (2.8)	19; 0; 3; 23
	All	202	165.5 (6.8)	63.0 (9.8)	22.9 (3.1)	62; 14; 69; 57
Female	50s	47	158.1 (4.9)	55.4 (9.0)	22.2 (3.8)	5; 4; 29; 9
	60s	61	152.8 (5.4)	52.2 (7.6)	22.3 (2.8)	21; 4; 17; 19
	70s	53	149.8 (5.2)	50.7 (8.0)	22.5 (3.2)	16; 3; 8; 26
	80s	48	144.6 (5.9)	48.3 (7.9)	23.1 (3.3)	11; 0; 5; 32
	All	209	151.4 (7.1)	51.6 (8.4)	22.5 (3.3)	53; 11; 59; 86

Notes: Values represent the mean (standard deviation). Primary industry jobs included agriculture and forestry. Secondary industry jobs involved manufacturing and construction. Tertiary industry jobs included food service and education. Abbreviations: BMI, body mass index; Pri, primary industry; Sec, secondary industry; Ter, tertiary industry.

**Table 2:** Tabulation results of spine parameters and SF8^TM^ summary scores.

Age (Years)	Number	CSVA (mm)	CL (deg.)	T1S (deg.)	SVA (mm)	Presence of Spondylosis	PCS (Points)	MCS (Points)
Male								
50s	50	23.1 (13.9)	10.5 (10.3)	25.3 (5.9)	5.8 (25.8)	66.0%	50.2 (6.4)	49.0 (6.0)
60s	53	28.4 (15.0)	9.1 (11.1)	27.3 (8.4)	9.1 (37.9)	69.8%	50.1 (7.0)	49.5 (5.3)
70s	54	29.1 (12.1)	13.3 (12.1)	28.4 (8.5)	21.7 (30.5)	88.9%	46.8 (7.0)	50.5 (5.3)
80s	45	30.8 (17.0)	13.6 (15.1)	31.2 (9.9)	56.7 (48.6)	100.0%	43.6 (8.7)	52.0 (6.8)
All	202	27.8 (14.7)	11.6 (12.2)	28.0 (8.4)	22.2 (40.9)	80.7%	47.8 (7.7)	50.2 (5.9)
Female								
50s	47	17.8 (10.9)	8.6 (10.4)	22.8 (6.9)	−5.4 (26.3)	46.8%	50.7 (5.6)	46.6 (7.1)
60s	61	15.6 (7.7)	8.7 (9.1)	22.1 (7.2)	4.7 (29.5)	67.2%	50.1 (5.8)	50.1 (4.8)
70s	53	17.4 (10.1)	13.3 (11.0)	25.1 (10.3)	31.2 (36.3)	75.5%	46.5 (7.5)	49.9 (6.1)
80s	48	18.6 (15.6)	19.2 (12.2)	30.1 (13.6)	60.9 (59.7)	89.6%	42.0 (8.7)	50.7 (6.8)
All	209	17.2 (11.2)	12.3 (11.4)	24.8 (10.2)	22.1 (46.6)	69.9%	47.5 (7.7)	49.4 (6.3)

Note: Values represent the mean (standard deviation). Abbreviations: CSVA, C2-C7 sagittal vertical axis; CL, C2-C7 cervical lordosis; T1S, T1 slope; SVA, sagittal vertical axis; PCS, SF-8^TM^ physical component summary score; MCS, SF-8^TM^ mental component summary score.

**Table 3:** Relationship between subcervical alignment and cervical alignment parameters with and without age adjustment.

	Crude		Age-Adjusted	
	Correlation Coefficient	*p*-Value	Correlation Coefficient	*p*-Value
Male				
SVA and CSVA	0.31	<0.01 *	0.25	<0.01 *
SVA and CL	0.22	<0.01 *	0.20	<0.01 *
Female				
SVA and CSVA	0.10	0.15	0.07	0.31
SVA and CL	0.27	<0.01 *	0.09	0.19

Note: * denotes a significant difference (*p* < 0.05). Abbreviations: SVA, sagittal vertical axis; CSVA, cervical sagittal vertical axis; CL, cervical lordosis.

**Table 4:** Effects of cervical alignment parameters on SF-8^TM^ physical component summary scores.

	Crude		Age-Adjusted	
	Effect	*p*-Value	Effect	*p*-Value
Male				
CSVA (+10 mm)	0.0 ± 0.4	0.98	0.3 ± 0.4	0.43
T1S-CL (+10 degrees)	−0.9 ± 0.5	0.08	−0.8 ± 0.5	0.08
SVA (+10 mm)	−0.5 ± 0.1	<0.01 *	−0.3 ± 0.1	0.04 *
Female				
CSVA (+10 mm)	−1.4 ± 0.5	<0.01 *	−1.2 ± 0.4	<0.01 *
T1S-CL (+10 degrees)	−0.5 ± 0.6	0.33	−1.0 ± 0.5	0.04 *
SVA (+10 mm)	−0.7 ± 0.1	<0.01 *	−0.4 ± 0.1	<0.01 *

Notes: Effect values represent the mean ± standard error. * Denotes a significant difference (*p* < 0.05). Abbreviations: CSVA, C2–C7 sagittal vertical axis; T1S-CL, T1 slope minus C2–C7 cervical lordosis; SVA, sagittal vertical axis.

**Table 5:** Effects of cervical alignment parameters on SF-8^TM^ mental component summary scores.

	Crude		Age-Adjusted	
	Effect	*p*-Value	Effect	*p*-Value
Male				
CSVA (+10 mm)	0.0 ± 0.3	0.90	−0.1 ± 0.3	0.74
T1S-CL (+10 degrees)	−0.2 ± 0.4	0.62	−0.2 ± 0.4	0.53
SVA (+10 mm)	0.2 ± 0.1	0.06	0.1 ± 0.1	0.48
Female				
CSVA (+10 mm)	−0.5 ± 0.4	0.21	−0.5 ± 0.4	0.22
T1S-CL (+10 degrees)	−0.7 ± 0.5	0.12	−0.6 ± 0.5	0.21
SVA (+10 mm)	0.1 ± 0.1	0.33	0.0 ± 0.1	0.68

Note: Effect values represent the mean ± standard error. Abbreviations: CSVA, C2–C7 sagittal vertical axis; T1S-CL, T1 slope minus C2–C7 cervical lordosis; SVA, sagittal vertical axis.

## Data Availability

The complete database of the cohort can be accessed at the Zenodo repository (doi.org/10.5281/zenodo.5723125).
